# Extremely Low Genetic Diversity Indicating the Endangered Status of *Ranodon sibiricus* (Amphibia: Caudata) and Implications for Phylogeography

**DOI:** 10.1371/journal.pone.0033378

**Published:** 2012-03-12

**Authors:** Shao-Yu Chen, Yi-Jun Zhang, Xiu-Ling Wang, Jian-Yun Sun, Yan Xue, Peng Zhang, Hui Zhou, Liang-Hu Qu

**Affiliations:** 1 State Key Laboratory of Biocontrol, Key Laboratory of Gene Engineering of the Ministry of Education, Sun Yat-sen University, Guangzhou, People's Republic of China; 2 Department of Biology, Xinjiang Normal University, Xinjiang, People's Republic of China; Institut de Biologia Evolutiva - Universitat Pompeu Fabra, Spain

## Abstract

**Background:**

The Siberian salamander (*Ranodon sibiricus*), distributed in geographically isolated areas of Central Asia, is an ideal alpine species for studies of conservation and phylogeography. However, there are few data regarding the genetic diversity in *R. sibiricus* populations.

**Methodology/Principal Findings:**

We used two genetic markers (mtDNA and microsatellites) to survey all six populations of *R. sibiricus* in China. Both of the markers revealed extreme genetic uniformity among these populations. There were only three haplotypes in the mtDNA, and the overall nucleotide diversity in the mtDNA was 0.00064, ranging from 0.00000 to 0.00091 for the six populations. Although we recovered 70 sequences containing microsatellite repeats, there were only two loci that displayed polymorphism. We used the approximate Bayesian computation (ABC) method to study the demographic history of the populations. This analysis suggested that the extant populations diverged from the ancestral population approximately 120 years ago and that the historical population size was much larger than the present population size; i.e., *R. sibiricus* has experienced dramatic population declines.

**Conclusion/Significance:**

Our findings suggest that the genetic diversity in the *R. sibiricus* populations is the lowest among all investigated amphibians. We conclude that the isolation of *R. sibiricus* populations occurred recently and was a result of recent human activity and/or climatic changes. The Pleistocene glaciation oscillations may have facilitated intraspecies genetic homogeneity rather than enhanced divergence. A low genomic evolutionary rate and elevated inbreeding frequency may have also contributed to the low genetic variation observed in this species. Our findings indicate the urgency of implementing a protection plan for this endangered species.

## Introduction

Loss of genetic diversity has been considered a crucial genetic factor that tends to produce inbreeding depression, reduced adaptation and fitness and a decrease in the long-term species survival [1], [2]. The assessment of the genetic diversity of populations is important for planning conservation strategies for endangered species, and it is often necessary to investigate the causes of low levels of genetic variation in populations. Many studies have shed light on the discrimination that whether the genetic uniformity has resulted from the effect of recent demographic and environmental pressures (e.g., in populations of the Javan rhinoceros [3], the Hawaiian monk seal [4] and the Galápagos penguin [5]), as opposed to the much older historical influence of Pleistocene climatic fluctuations (e.g., in populations of the Iberian lynx [6], the Madagascar fish-eagle [7] and killer whales [8]). Such studies have employed many approaches to distinguish between these two scenarios, e.g., utilizing historical (museum) samples to compare the genetic variation before and after the decline [6], or using different types of analytical methods (e.g., coalescent-based modeling [7] and Bayesian clustering [9]) to test the historical and contemporary gene flow or effective population size (*N*_e_) based on extant samples. And it should be stressed that molecular markers with different rates of substitution can capture signatures of evolutionary processes at different timescales and can therefore greatly influence the interpretations of historical demographic patterns [10]–[12]. Studies that utilize a particular marker exclusively should consider the limitations of that marker, particularly the timescale of the evolutionary processes that it captures, and should be cautious in making conclusions regarding recent versus historical processes. The use of different markers in combination will provide a better idea of the actual genetic variation and a more accurate picture of the historical evolutionary events. A recent review suggests that more than 70% of the papers use only mtDNA markers in the study of mammals, amphibians and birds, whereas the proportion of combination of two types of genetic markers were only 5% [13]. Amphibian populations, which provide a good model for studies of conservation genetics and phylogeography, are well known to be declining dramatically on a global scale [14], [15]. In contrast to mammals, fishes and birds, which have strong dispersal ability, amphibians are more prone to become endangered when facing unfavorable conditions because of the characteristics of limited dispersal ability, strong site fidelity and fragmented breeding habitat [16], [17]. Especially, the montane and stream-breeding amphibian species face a particularly high risk of extinction [16], [18]. This most-endangered status makes exact and comprehensive evaluation of genetic variation of these species urgent and imperative.

**Figure 1:**
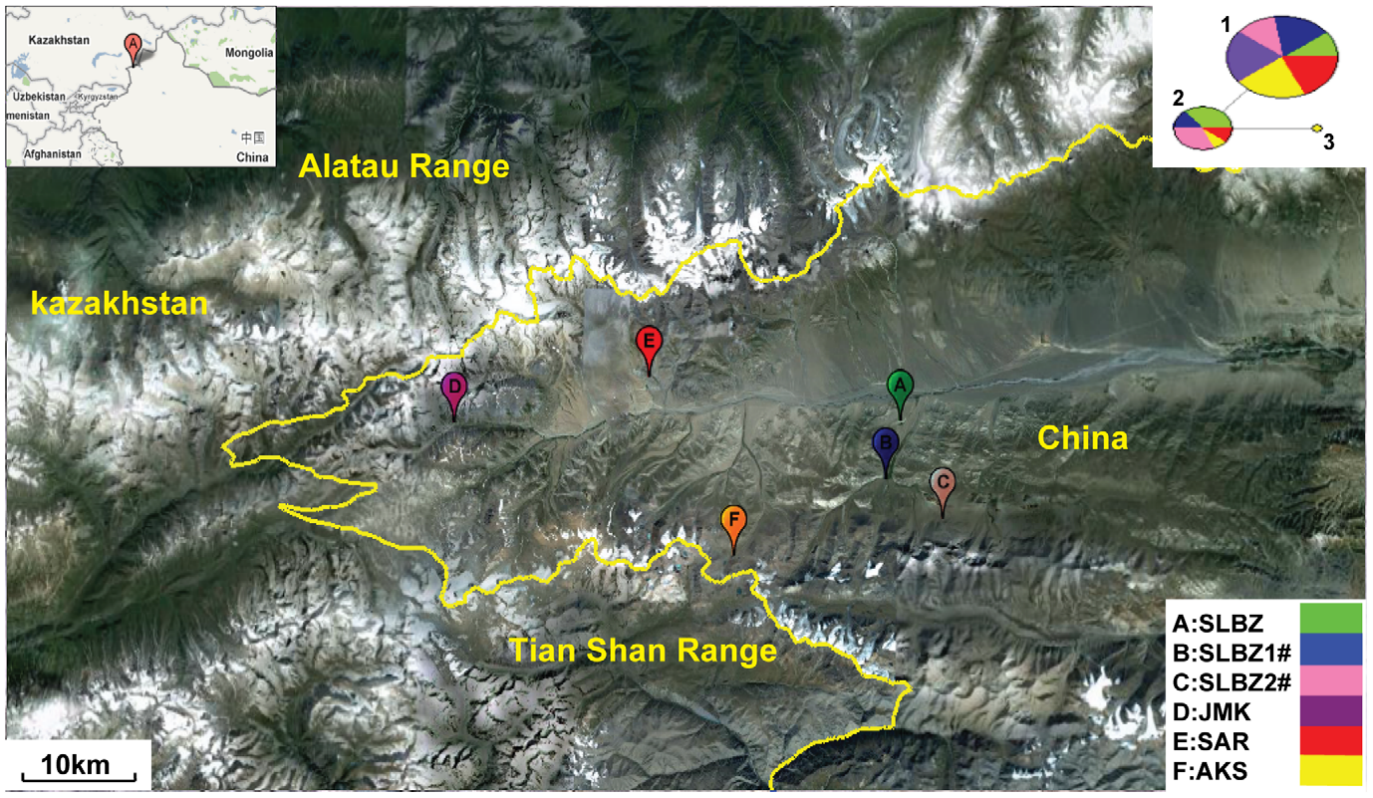
Map of six *R. sibiricus* populations in China and the MJ network analysis of mtDNA. These populations are located at Tian Shan (Biezhentao Range) and Junggarian Alatau, and their characteristics are listed in Table 1. The dark and white areas on the map indicate low altitude and ice-covered mountaintops, respectively. The top right corner shows the MJ network analysis of the relationships between the three haplotypes of mtDNA. The circled area is proportional to the haplotype frequency and each color represents a population (bottom right corner).

The Siberian salamander (*Ranodon sibiricus*; Amphibia: Caudata) is a montane species that is well suited for such studies. This species is endemic to two mountains of Central Asia (the Junggarian Alatau and the Northern Tian Shan) and is restricted to geographically small areas in Wenquan County, northwestern Xinjiang of China and southern Kazakhstan. Only six small and isolated populations have been detected in China (Figure 1) [19], [20]: Sulbeijin (SLBZ), Sulbeijin #1 (SLBZ1#), Sulbeijin #2 (SLBZ2#), Aksai (AKS), Zemaike (JMK) and Sarbastou (SAR). In recent decades, the condition of wild *R. sibiricus* has become increasingly serious as a result of habitat deterioration and anthropogenic activities [21]. In particular, wetlands, which are very important for the survival of amphibians [22], [23], have greatly receded because of dry weather in recent years. The populations of wild *R. sibiricus* have consequently decreased in a continuous and dramatic manner in all of the regions where they are distributed [19], [24], [25]. *R. sibiricus* has been listed as “Endangered” on the International Union for Conservation of Nature (IUCN)'s red list of threatened species for Kazakhstan and as “Critically Endangered” on the red list of threatened species for China (2005). Although various aspects of this species' ecology, morphology, physiology, cytogenetics and phylogenetic position have been well documented, there are few data on the genetic variation within and among populations in China [26]. The evaluation and characterization of regional population genetic structure and population processes of this species are therefore of immediate importance and will provide useful information on population status and demographic history that will greatly improve recovery plans [27].

Many recent studies have focused on the influence of topography and Pleistocene climatic changes on regional species [28], [29]; however, the demographic history of many species in the mountain ranges of Central Asia remains poorly understood [30], [31]. Climatic oscillations in montane regions can cause species to expand or contract along elevational gradients [32], [33]. It has been commonly observed that alpine species always expanded during inter-glacial periods, which facilitated gene flow among populations; whereas species contracted or survived *in situ* during the coldest phases of the glacial periods, which may have lead to genetic divergence [30], [34]. However, the way that species responded to climatic oscillations may be different, especially for amphibians. Researchers should take the characteristics of species into account, including behavioral and ecological attributes [35]–[37]. As a result of the uplift of the northern Tian Shan and the Junggarian Alatau, *R. sibiricus* is thought to have become a relict species in unglaciated regions (i.e., streams running out of ice-capped mountains) at altitudes of approximately 2100–3200 m above sea level, where the habitat is at almost the extreme limit of tolerance for cold-blooded animals. To adapt to its unique environment, *R. sibiricus* has developed some behavioral and ecological traits that have presumably been caused by selection pressures. For example, its egg sacs favor cold stream water; its larvae develop slowly; and its hibernation time is as long as half a year [21], [38]. There were at least four main Quaternary glaciations in the Tian Shan and the Junggarian Alatau after the uplift of the Tibetan plateau [39]. The six populations of *R. sibiricus* in China were located at different altitudes and isolated far from each other. An interesting question is whether these six populations survived the Quaternary ice ages *in situ* or responded to climatic oscillations in a similar manner to other alpine species.

In this study, we utilized two types of markers, mitochondrial DNA (D-loop region) and microsatellites, to characterize the patterns of genetic diversity in all the populations of *R. sibiricus* in China. We then attempted to determine whether those patterns are due to recent environmental impacts or due to much older historical events (e.g., Pleistocene climatic fluctuations), using statistical evolutionary hypotheses under the approximate Bayesian computation (ABC) method. The ABC method has exhibited powerful potential in phylogeography [40]–[42], particularly in cases of low genetic diversity that are difficult to analyze using other methods. The results of the present study will help advance our understanding of the population history of endangered species, particularly those with limited genetic data.

## Materials and Methods

### Ethics statement

The tissue samples were collected under the permission of the administrative institution of the Forest Department of Xinjiang Province, which is responsible for the regulation of local animals in Xinjiang Province. Our project was also approved by the National Natural Science Foundation of China (No. 30771151) and supported by Sun Yat-sen University.

### Sample collection and DNA extraction

We collected 123 samples across all six known localities of *R. sibiricus* in China, from May to August 2006 (Table 1). Samples from the SLBZ1# and JMK sites were collected by Zhang Yijun and Xue Yan; samples from the other sites were collected by Wang Xiuling. Three samples from AKS collected in August 2005 had six toes on the hind limbs. Tissue samples were collected from each animal by removing approximately 5–10 mm of the tail using sterile equipment, and the animals were subsequently released. The tissue samples were stored in 95% ethanol at −70°C and the DNA was later extracted using the Qiagen DNeasy Tissue Kit (Qiagen) according to the manufacturer's protocol.

**Table 1:** The habitat and population size data, sampling time, sampling size and sample size of each locality used for (a) mtDNA and (b) microsatellite analysis.

Locality	Discovered time	Coordinates	Area(m^2^)	Altitude (m)	Population size existed	Sampling time	Sampling size	Sample size	
								a	b
SLBZ	1990	N: 44°56′04, E: 80°30′43	40000	2100–2300	>1000	May 2006	20	20	0
SLBZ1#	1991	N: 44°53′27, E: 80°29′45	10000	2041	120	June 2006	20	20	20
SLBZ2#	2006	N: 44°50′01, E: 80°39′10	30000	2250	150	August 2006	20	20	20
JMK	1989	N: 43°55′47, E: 80°00′18	5000	2800	100	June 2006	20	20	20
SAR	2002	N: 44°57′59, E: 80°13′34	15000	2465	120	May 2006	20	20	20
AKS	1996	N: 44°49′53, E: 80°19′26	10000	3200	1500	August 2006	23*	23	20

*Three samples of AKS (21, 22 and 23) obtained by Xiuling Wang in August 2005 had six toes on the hind limbs.

### mtDNA sequencing

We aligned the entire mtDNA genome of seven genera of the family Hynobiidae (*Batrachuperus*, *Hynobius*, *Liua*, *Onychodactylus*, *Pachyhynobius*, *Ranodon* and *Salamandrella*) and found that the D-loop region was the most variable region. We therefore chose a segment of mtDNA that encompassed the D-loop region to investigate the genetic variability within and among populations of *R. sibiricus*. A 1323 bp segment of the mitochondrial genome was selected that spans the tRNA-Thr, tRNA-Pro, tRNA-Phe, D-loop region and partial Cyt*b* and 12S rRNA sequences and has been found to show considerable variation [43]. The primers Cyt*b* A [44] and 12S71R were used (Table S1). All 123 samples of the six populations were amplified using Prime polymerase (TaKaRa), and the PCR products were then purified using the QIAquick PCR Purification Kit (Qiagen) and sequenced in both directions.

### Microsatellite characterization

Microsatellites are an ideal biparentally inherited nuclear marker with high resolution for estimating male-mediated gene flow and population structure [11]. Many studies have demonstrated the power of using the combination of both nuclear DNA and mtDNA in assessing genetic structure in conservation and phylogeography [45]–[47]. Microsatellite enrichment was performed according to the protocols of Glenn and Schable [48]. Two types of probes, (CA)_13_ and (GATA)_8_, were used to construct the libraries. The colonies were screened for inserts using a technique similar to the PIMA method [49], with an M13 forward primer and nonbiotinylated oligo (CA)_10_ or (GATA)_5_ as primers. The clones produced one clear band in agarose electrophoresis that was likely to contain microsatellite repeats. The positive clones with distinct amplification bands were obtained and sequenced. The sequences were proofread by eye with the aid of the Tandem Repeats Finder software [50]. The flanking regions of the sequenced clones that contained unique repeats were chosen to design primers using the Primer Premier 3.0 program [51]. Initially, the PCR products of primer sets containing bands of expected-size and repeatable banding patterns were chosen to test the polymorphism between populations, with each population represented by eight samples. The PCR conditions were optimized in a gradient thermocycler (iCycler, Bio-Rad). The PCR products were separated on an 8% non-denaturing polyacrylamide gel at 200 V for 3–4 h and visualized by staining with ethidium bromide. Product sizes were estimated by comparison with the Pbr322/MspI DNA ladder and sized using Quantity One (Bio-Rad). Then we used 20 individuals from each of five populations (SLBZ1#, SLBZ2#, AKS, JMK, SAR), for a total of 100 individuals to evaluate the genetic characteristics of the polymorphic loci.

### Data analysis

#### Genetic diversity and population structure analyses

The sequences obtained from the two markers were edited and aligned by eye using the DNAStar 5.0 program. For comparisons of within-region genetic diversity, DnaSP4.0 was used to estimate population haplotype diversity, mean number of pairwise differences and nucleotide diversity. Neutrality tests of Tajima's *D* [52] and Fu's *Fs* [53] were calculated using Arlequin version 3.1 [54]. Significant values derived from either Tajima's *D* or Fu's *Fs* indicate that the sequences deviate from neutrality or that populations were previously subdivided and/or have experienced past fluctuations (i.e., are not in migration-drift equilibrium). The genetic differentiation and gene flow between populations were also assessed, by calculating pairwise *F*_ST_ values using the same software. Contrasting plots of observed vs. theoretical distributions of site differences (mismatch) gave insight into past population demographics [55], [56]. A median-joining network (MJ) [57] was used to visualize the relationships among haplotypes of mtDNA. The MJ network was constructed using the NETWORK4 program (www.fluxus-engineering.com). The TCS program, version 1.21 [58] was also used to create a network of corrected mtDNA haplotypes using the 95% statistical parsimony method of Templeton et al. [59]. Tests for observed heterozygosity (*H*_O_), expected heterozygosity (*H*_E_) and F-statistics were performed using the POPGENE 32 program, version 1.32 [60]. Tests for Hardy-Weinberg equilibrium (HWE) and linkage disequilibrium were performed using the GENEPOP 3.4 program [61] for microsatellite loci.

#### Approximate Bayesian computation analysis

**Figure 2:**
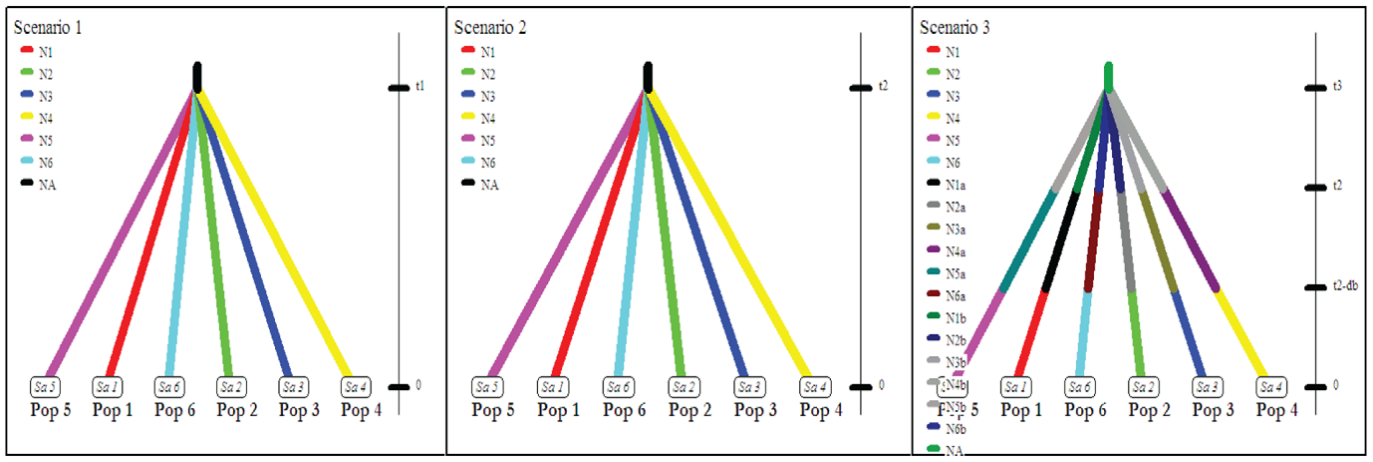
Three putative scenarios explored with ABC method. In all scenarios, the time is not to scale. See the text and supplementary materials (Text S1, Tables S2 and S4) for details.

To reconstruct the evolutionary history among these six populations of *R. sibiricus*, we applied the approximate Bayesian computation (ABC) method using the DIYABC program (version 1.0.4.39) [62]. This program allowed us to combine different markers (e.g., mtDNA and microsatellite) to compare putative evolutionary scenarios without explicit likehood calculations. An evolutionary scenario is established on the basis of certain parameters, including the times of events, effective population sizes and admixture rates. Through the comparison of different putative scenarios, the best-supported scenario can be accepted and used to explain the observed genetic data. We sought to compare three specific scenarios according to the genetic data we obtained and to test the putative evolutionary processes underlying this genetic pattern (Figure 2 and Table S2). The three scenarios were as follows: (1) the isolation of *R. sibiricus* populations was caused by recent habitat deterioration and anthropogenic activities; (2) *R. sibiricus* populations have been separated for a long time; individual populations merged during the Pleistocene glaciations and became separated again following the last glaciation; (3) *R. sibiricus* populations became separated before the Pleistocene glaciation oscillations, and the glaciations caused a bottleneck effect on individual populations rather than facilitating the mixing of populations.

In view of the limited dispersal ability and isolated geographic localities of the six populations, each population was treated as having evolved independently. In all of our scenarios, the six populations (AKS, JMK, SAR, SLBZ, SLBZ1# and SLBZ2#) had potentially different contemporary *N*_e_ value (N1, N2, N3, N4, N5 and N6, respectively). According to the recently observed population sizes (Table 1), the prior distribution of *N*_e_ for each of these populations was set up as between 1 to 500, 1 to 100, 1 to 100, 1 to 500, 1 to 100 and 1 to 100, respectively, based on an average estimation of vertebrate *N*_e_/*N*_C_ ratio of 0.38 [63]. In Scenarios 1 and 2, all populations diverged independently at times t1 and t2, respectively, from an ancestral population characterized by its own effective population size (NA). In Scenario 1, all populations were assumed to have become isolated recently from a large ancestral population, and t1 was set between 1 to 1000 generations looking backward in time, i.e., from the present to 5000 years before the present (YBP) assuming a generation time of 5 years [64]. This selection represents the timescale of recent human and environmental impacts. In Scenario 2, all populations were assumed to have diverged from a mixed population that arose during the Pleistocene glaciations, and t2 was set between 2000 to 8000 generations, i.e., from 10000 to 40000 YBP, which covers the timescale of the Pleistocene climatic fluctuations. In Scenario 3, we considered another effect of glaciations on population dynamics by introducing a set of parameters related to the bottleneck event for each population that occurred at time t2. The time db (the bottleneck events occurring in the first few generations following introductions or survived glacial periods) was set for 5 generations, and each population had a larger *N*_e_ (e.g., N1b, 10 to 1000) than after the bottleneck occurred (e.g., N1a, 1 to 100). The six populations were considered to have diverged from an ancestral population at a much earlier time (t3), which was set between 10000 to 50000 generations. The NA values of all three scenarios were considered to be identical, between 10 to 10000, based on the assumption that historical population sizes were much larger than present population sizes. This is according to the fact that the population sizes observed at the time the populations were first discovered were larger than today (e.g., for the SLBZ population). For the three scenarios, we ordered the times as t1<t2<t3. The other parameters are listed in Text S1. Under these scenarios, we wished to test whether the observed genetic structure of *R. sibiricus* was caused by recent human and environmental factors (Scenario 1) or by historical climatic oscillations (Scenario 2 and 3). If the latter was the case, we wished to test whether the effect of glaciations on population dynamics is to increase the gene flow among populations (Scenario 2) or to reduce the size of separated populations (Scenario 3).

## Results

### Isolation of microsatellites from *R. sibiricus*

Over 3000 clones of the CA and GATA libraries were screened in a search for microsatellites, and 130 positive clones having one distinct amplification band were obtained and sequenced. Of these 130 sequenced clones, 70 contained unique repeat sequences, of which the numbers of mono-, di-, tri-, tetra-, and pentanucleotide repeat sequences were 7, 50, 7, 5 and 1, respectively. We designed 66 primer sets from the flanking regions of 59 loci (for some loci that were difficult to amplify, we designed more than two sets), of which 50 contained expected-size bands and repeatable banding patterns and 9 were not amplified consistently. Initially, these 50 loci were tested for polymorphism with a small sample pool. Only 2 loci, Rsi-5 and Rsi-17, showed microsatellite polymorphism. To confirm this result, of the 48 loci without polymorphism, 15 loci with more than 10 dinucleotide repeats and 5 loci with more than 5 tetranucleotide repeats (Table S3; GenBank Accession numbers JN863391-JN863412) were retested for polymorphism using 40 samples. The result showed that these loci were still monomorphic. One hundred individuals from five populations were used to evaluate the genetic characteristics of the 2 polymorphic loci Rsi-5 and Rsi-17.

### Genetic diversity of *R. sibiricus* revealed by two types of markers

The mtDNA sequencing of 123 samples with PCR products yielded an average length of 1231 bp, and there were no sites with alignment gaps or missing data. Surprisingly, we detected a total of only 4 variable sites (Table 2). The levels of nucleotide and haplotype diversity were extremely low. The nucleotide diversity of *R. sibiricus* populations was 0.00064, ranging from 0.00000 (JMK) to 0.00091 (SLBZ) (Table 3). There were only 3 haplotypes overall (GenBank Accession numbers JN863413-JN863415), of which Hap-1 and Hap-2 were present in 94 and 28 samples, respectively, whereas Hap-3 occurred only in one specimen of the AKS population (Table 2).

**Table 2:** The haplotypes in the mtDNA of 123 samples.

	1 1 1 1							
	0 0 0 0							
	4 4 6 6							
	8 9 5 6	AKS	JMK	SLBZ	SLBZ1#	SLBZ2#	SAR	Frequency
Hap-1	GAAG	20	20	10	16	11	17	94
Hap-2	AGAG	2	0	10	4	9	3	28
Hap-3	GAGA	1						1

**Table 3:** Results of demographic parameters of mtDNA data.

Region	n	n_H_	Haplotype diversity, h	Nucleotide diversity, π(±95% CI)	Fu's *Fs*	Tajima's *D* (*P*-value)
AKS	23	3	0.245	0.00044	8.212(0.995)	−1.562(0.050)
JMK	20	1	0.000	0.00000	0.000(N.A.)	0.000(1.000)
SLBZ	20	2	0.526	0.00091	15.100(1.000)	2.923(1.000)
SLBZ1#	20	2	0.337	0.00058	11.228(0.999)	0.657(0.769)
SLBZ2#	20	2	0.521	0.00090	15.004(1.000)	2.860(1.000)
SAR	20	2	0.268	0.00046	9.568(0.998)	−0.161(0.474)
Overall	123	3	0.367	0.00064	19.037(0.997)	1.323(0.917)

Including number of populations, *h*, *π*, Fu's *Fs*, Tajima's *D* (*P*-values given where applicable).

n, number of samples; n_H_, number of haplotypes. Fu's *Fs* test cannot be computed in JMK which contained only one allele in samples.

For the two polymorphic loci of the microsatellites, the number of alleles was two per locus. The *H*_O_ and *H*_E_ values of these loci ranged from 0.080 to 0.090 and from 0.077 to 0.086, respectively (Table 4). One allele of the Rsi-5 locus existed only in the SLBZ2# and JMK populations and was particularly abundant in the JMK population. No locus showed significant deviation from HWE or significant linkage disequilibrium. In the genomic DNA of vertebrates, (CA)_n_ is the most common dinucleotide motif, comprising a proportion of 30–67%, and higher-order SSR classes (tri-, tetra-, penta- and hexanucleotides) are less common than dinucleotides [65]. According to Gao et al. [66], the average number of alleles of microsatellites containing CA repeats in the range from 10 to 20 is 9.2 in amphibians and reptiles. However, in the present study, the 15 loci with more than 10 CA repeats were all monomorphic. In contrast, the two loci that showed polymorphism were of tetranucleotide repeats but not of dinucleotide repeats.

**Table 4:** For each locus of polymorphic microsatellites, the number of individuals (n) genotyped for each population, expected level of heterozygosity (*H*_E_), observed level of heterozygosity (*H*_O_) and *F*_IS_, *F*_ST_ –value.

	SLBZ1# (n = 20)			SLBZ2# (n = 20)			AKS (n = 20)			JMK (n = 20)			SAR (n = 20)			All sites		
Locus	*H*_E_	*H*_O_	*F*_IS_	*H*_E_	*H*_O_	*F*_IS_	*H*_E_	*H*_O_	*F*_IS_	*H*_E_	*H*_O_	*F*_IS_	*H*_E_	*H*_O_	*F*_IS_	*H*_E_	*H*_O_	*F*_ST_
Rsi-5	0.000	0.000	0.000	0.050	0.050	−0.026	0.000	0.000	0.000	0.296	0.350	−0.212	0.000	0.000	0.000	0.077	0.080	0.121
Rsi-17	0.050	0.050	−0.026	0.050	0.050	−0.026	0.097	0.100	−0.053	0.050	0.050	−0.026	0.185	0.200	−0.111	0.086	0.090	0.020

Allelic data and genetic variation were derived from 100 specimens of *R. sibiricus* from five populations in China. There were no missing genotyped individual in those populations.

### Population structure and demographic analyses of *R. sibiricus*

**Figure 3:**
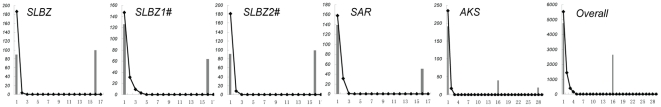
Mismatch distributions of mtDNA of the populations. The histogram shows the observed distribution of pairwise differences between the populations (excluding JMK, which showed only one haplotype) and these populations treated as a whole (*Overall*). The black line with diamonds shows the expected distribution for a growing population under a population expansion model.

Although the levels of haplotype and nucleotide diversity of mtDNA were very low, we found that the haplotype Hap-2 was relatively abundant in SLBZ and SLBZ2# and was absent in JMK. Therefore, the analysis of pairwise differences among these three populations showed that JMK was significantly differentiated from the other two (Table 5). Neutrality tests showed significant positive values of Tajima's *D* and Fu's *Fs* test for most of the populations, indicating that the populations had experienced past fluctuations and were not in migration-drift equilibrium. Only AKS and SAR, which contained mainly the haplotype Hap-1, showed low negative values of Tajima's *D* without obvious statistical significance (Table 3). The low levels of nucleotide diversity precluded meaningful phylogenetic reconstruction using the MJ network (Figure 1) and TCS analysis (data not shown). Because of the lack of sufficient data on haplotypes, pairwise mismatch distributions for five populations and for all populations treated as a whole showed no clear indication of either recent demographic expansion or demographic contraction (Figure 3).

**Table 5:** Results of pairwise differences within and among populations.

	SAR	JMK	SLBZ1#	SLBZ2#	SLBZ	AKS
SAR	---	---	---	---	---	---
JMK	0.1053	---	---	---	---	---
SLBZ1#	−0.0436	0.1579	---	---	---	---
SLBZ2#	0.1511	0.4211	0.0874	---	---	---
SLBZ	0.2053	0.4737	0.1368	−0.0474	---	---
AKS	−0.0358	0.0483	−0.0180	0.1885	0.2414	---

Neutrality tests and mismatch analysis are not effective tools for revealing the historical demography of populations [2], especially in cases of extremely low genetic diversity such as the one under study. We therefore used the ABC method to distinguish between the three competing evolutionary scenarios based on our data (Figure 2). The result showed that Scenarios 2 and 3 were statistically rejected (probability = 0.0001), whereas Scenario 1 was strongly supported (probability = 0.9999) (Table 6). In view of this result, we inferred the posterior distribution of parameters for Scenario 1 only (Table 7). Because the parameters did not show obvious bias or dispersion, we drew point estimates toward the mean values of prior distributions. Bias and precision of parameter estimation under Scenario 1 were listed in Table S4. We concluded that the six extant populations diverged 24 generations ago (the upper value of the 95% CI was 57) from the common ancestral population, i.e., 120 YBP (the upper value of the 95% CI was 285) assuming a generation time of 5 years [64]. The small value of the divergence time (24 generations) suggests that these populations were isolated from each other at a recent historical time, when they were subject to strong disturbances from human activities and/or environmental changes. The estimated effective population sizes (*N*_e_) for the six populations were 278, 54, 60, 108, 68 and 64, respectively. The NA value of the common ancestral population is roughly two orders of magnitude higher than that of the extant populations (NA = 2640), suggesting the occurrence of a dramatic historical decline in the number of individuals.

**Table 6:** Relative posterior probabilities with 95% credibility intervals for each scenario.

Historical scenario	30,000 simulations
Scenario 1	0.9999 [0.9999–0.9999]
Scenario 2	0.0001 [0.0001–0.0001]
Scenario 3	0.0000 [0.0000–0.0000]

The logistic regression used to compute posterior probabilities considered the 30,000 simulated data sets closest to the observed data (1% of the total number of simulations performed for the three scenarios, respectively).

**Table 7:** Properties of the approximate posterior distribution of parameters under Scenario 1.

Parameter	mean	median	mode	Q_0.025_	Q_0.050_	Q_0.950_	Q_0.975_
N1	2.78E+002	2.80E+002	2.57E+001	5.13E+001	7.31E+001	4.76E+002	4.89E+002
N2	5.37E+001	5.31E+001	9.60E+001	9.60E+000	1.39E+001	9.46E+001	9.71E+001
N3	6.00E+001	6.14E+001	1.27E+001	1.27E+001	1.80E+001	9.62E+001	9.83E+001
N4	1.08E+002	6.72E+001	7.30E+001	7.30E+000	1.01E+001	3.51E+002	4.10E+002
N5	6.75E+001	7.11E+001	1.86E+001	1.86E+001	2.51E+001	9.78E+001	9.91E+001
N6	6.43E+001	6.69E+001	8.53E+001	1.67E+001	2.27E+001	9.71E+001	9.87E+001
t1	2.42E+001	1.31E+001	7.30E+000	2.80E+000	3.70E+000	5.71E+001	1.26E+002
NA	2.64E+003	1.87E+003	4.55E+002	2.21E+002	3.28E+002	7.70E+003	8.69E+003
Mμmic_A	2.44E−004	1.71E−004	1.14E−004	1.03E−004	1.05E−004	6.76E−004	8.22E−004
Mμseq_M	7.51E−008	8.09E−008	9.87E−008	2.28E−008	3.23E−008	9.87E−008	9.94E−008

Mean, median, mode and quantiles of the posterior distribution sample for original and composite parameters of the simulated data set. Results have been obtained with uniform priors on parameters.

## Discussion

### Genetic diversity and population structure of endangered *R. sibiricus*

We expected to find that the population structure of *R. sibiricus* was deeply differentiated as a result of the fragmented habitat, mountain barriers to gene flow, small population sizes and low dispersal capacity [67]–[69]. However, we observed low genetic diversity and divergence in the mtDNA marker among the populations and a low number of polymorphic loci in the microsatellites. Thus, the data from both these markers indicated an extreme depletion of genetic variation among the *R. sibiricus* populations.

**Figure 4:**
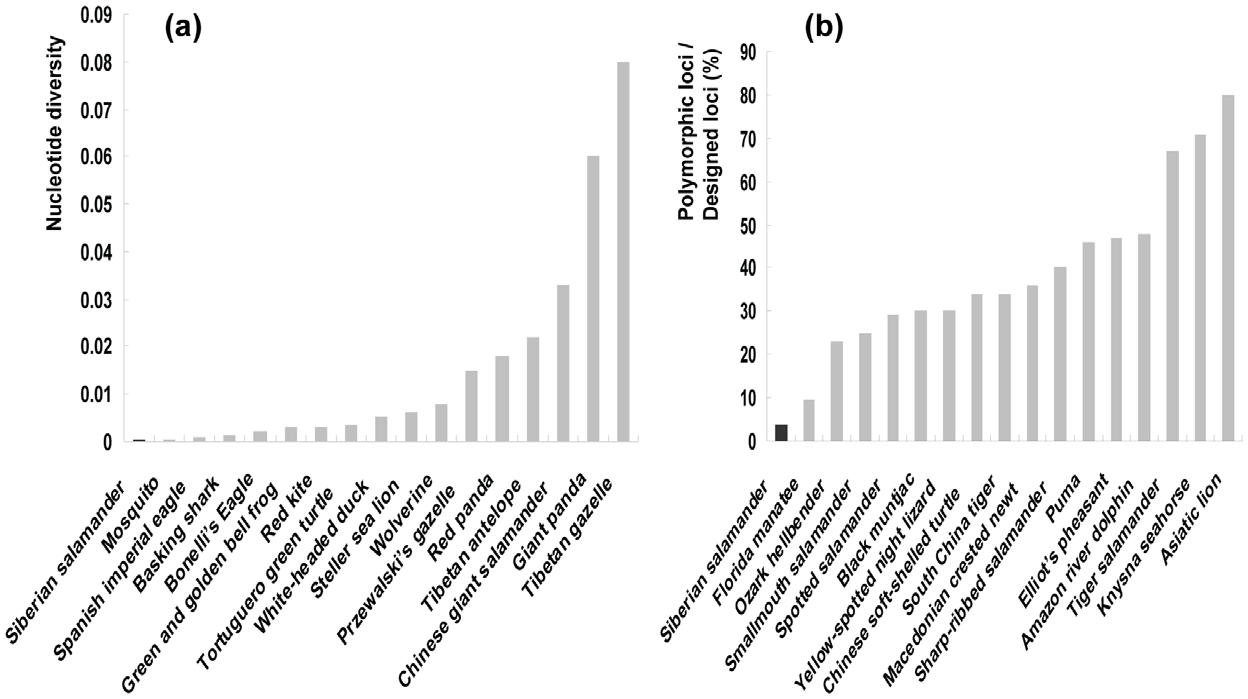
Histograms of comparisons with other species. (a) Nucleotide diversity of mtDNA compared with other endangered species. (b) Diversity of microsatellites (ratio between polymorphic loci and designed primer pairs of loci (%)) compared with other endangered species. The first species is *R. sibiricus*, indicated by a black box; other species are shown as gray boxes. The species in (a) and (b) are listed with common names. For references of the species, see Text S2.

This finding was consistent with a preliminary survey of *R. sibiricus* by Zhang *&* Wu [26], which used a 667-bp segment of the D-loop to investigate the genetic variation among four different populations with an overall total of 21 samples and found genetic uniformity among the populations. Such low genetic diversity is in strong contrast to published findings for many other amphibians and endangered animal species. A number of previous studies of hynobiid salamanders have shown high genetic diversity within a species and even within a single population [70], [71]. Genetic diversity data for mtDNA and microsatellites of a variety of endangered species are summarized in Figure 4. Of all the species studied, *R. sibiricus* showed the lowest genetic diversity for these two markers.

The uniformity of genetic markers for *R. sibiricus* appeared to be inconsistent with the morphological differences observed in some individuals; this inconsistency has also been observed in other amphibians [72], [73]. For example, some AKS individuals differed in having six toes on the hind limbs, but showed no difference in the two genetic markers studied. In most vertebrates, including other amphibians, the presence of extra digits has been associated with inbreeding [74], [75]. Given the low genetic diversity in *R. sibiricus*, it seems reasonable that the extra digit is a result of inbreeding, rather than a local adaptation to the surrounding environment (e.g., the flat area around the lake at the AKS site). Although some species display a low level of genetic diversity among populations, they display a strong phylogeographic structure [68], [76]–[78]. In our mtDNA study, the geographically adjacent SLBZ, SLBZ1# and SLBZ2# populations showed greater genetic similarity than other populations; e.g., the Hap-2 haplotype was distributed mainly in these three populations (Table 2). JMK, which is far from the other populations, showed only one haplotype. This finding is consistent with the principle that the degree of genetic differentiation is related to geographic proximity. Furthermore, the polymorphic microsatellite locus Rsi-5 showed high allelic frequencies in JMK individuals but little or no frequency in the other populations (Table 4). The data sets for mtDNA and microsatellites indicated that JMK was significantly differentiated from the other populations. We observed that the population size of JMK had declined to 100 or fewer individuals during our sampling (Table 1). We therefore inferred that the differentiation of this population probably resulted from genetic drift caused by population declines, which can quickly lead to a marked divergence in allelic frequencies [79]. The analysis of mtDNA and microsatellites in combination suggested that these populations showed a degree of shallow differentiation, although this conclusion seemed weakly supported. Our findings were based only on the populations of *R. sibiricus* in China. Future studies should include genetic data from the populations in Kazakhstan to determine whether those populations exhibit greater differentiation (relative to each other or to the China populations) or low genetic diversity similar to that of the China populations.

### Possible causes of low genetic diversity

Extremely low genetic diversity accompanied by shallow population differentiation was observed in this study. These findings can be attributed to several factors, including: (a) special population history, (b) a slow evolutionary rate at the genomic level and (c) a high frequency of inbreeding resulting from a small population size.

The Pleistocene glaciation cycles are considered to have been critical in shaping the distributions and genetic attributes of species [30], [34], [80]. During the glaciation extremes, species were forced into isolated refugia, thereby enhancing intraspecific differentiation. This is a commonly observed phenomenon for many terrestrial species that were unable to withstand the cold temperature conditions. However, for species that had become adapted to high-elevation environments, such as *R. sibiricus*, the glaciations may have had the opposite effect of enhancing the interactions among populations. This hypothesis is supported by the fact that *R. sibiricus* has evolved several traits to adapt to high-elevation habitats: (i) the egg sacs favor cold water in the headwaters of small mountain streams, brooks and lakes; (ii) the larvae develop slowly and feed mainly on stream invertebrates; and (iii) the hibernation time is as long as half a year [21], [38]. During the inter-glaciation periods, these traits enabled *R. sibiricus* populations to be scattered at altitudes approximately 2100–3200 m above sea level on different mountains, thus isolating the populations from one another. When glaciation occurred, the montane species expanded and contracted along an elevation gradient in response to the descending snow limit [32], [33]. This process provided opportunities for *R. sibiricus* to reach the feet of mountains, and the gene flow among previously isolated populations could be unblocked.

At least four main Quaternary glaciations occurred in the Tian Shan [39]. The early Pleistocene glaciation history of some central regions is not well understood [81]. During the Middle and Late Pleistocene, the permafrost of the northern and central plains of Kazakhstan merged with the permafrost of the mountains in the south [82]. During the Last Glacial Maximum, the permafrost came down to ∼900 m a.s.l. [82] in Kazakhstan but reached only ∼1800 m a.s.l. in the Tarim Basin [83]. *R. sibiricus* therefore had to move down ∼1000 m to the feet of mountains along streams, which facilitated the interactions between populations. The multiple glaciations may also have caused frequent gene flow among the populations.

The ABC method applied in this study gave no statistical support to Scenario 3, which assumed that the six populations diverged independently from a common ancestral population before the Pleistocene period and underwent a bottleneck process during the glaciations. This finding suggests that a glaciation effect leading to greater intraspecies divergence did not occur in *R. sibiricus*, as also evidenced by the shallow genetic structure among the populations. In contrast, intraspecific gene flow may have been facilitated during this period, as assumed in Scenario 2. A possible reason for the statistical rejection of Scenario 2 is our assumption that the separation of the *R. sibiricus* populations occurred shortly after the Last Glacial Maximum. Scenario 1, which assumed that the divergence happened within 1000 generations, was strongly supported by the ABC method, suggesting that the divergence occurred only 120 years ago, when the populations were under the strong influence of human disturbances and/or recent climatic changes (e.g., global warming). In this scenario, we proposed that the historical distribution of *R. sibiricus* was continuous and that the species may have consisted of a single large population until recently (e.g., until several hundred years ago). This scenario would imply that the distribution range of *R. sibiricus* was much wider at that time than at present. Recent human activities and climatic changes may have caused habitat fragmentation as well as population size decline. Recent grazing has been reported to cause great harm to *R. sibiricus* populations during the time of reproduction. In view of its limited motility, *R. sibiricus* is extremely vulnerable to the damage caused by herds of cattle; e.g., egg sacs in the streams are likely to be destroyed by the cattle. Recent studies have shown that global warming may have driven the extinction of amphibians by facilitating epidemic diseases [84]. We do not have evidence that the decline of *R. sibiricus* was caused by a particular pathogen, but this species is certainly vulnerable to pathogenic infection. In view of the cold-resistant adaptations of *R. sibiricus*, climatic warming may have cause shifts of the populations toward higher-altitude habitats, which could aggravate the fragmentation of populations.

Genomic evolutionary rates have been reported to vary depending on the species. For instance, mtDNA exhibits a low evolutionary rate in plants and some turtles [11], [76], [85]. In our study, the low observed genetic diversity of both mtDNA and nuclear DNA may have been due in part to the slow genomic evolutionary rate of *R. sibiricus*. There is no direct evidence to support this hypothesis because we could not find ancient samples to infer the genomic evolutionary rate of this species. However, a study by Allen et al. indicated that individual metabolic rate is a primary determinant of evolutionary rates: 10^13^ J of energy flux per gram tissue generates one substitution per nucleotide in the nuclear genome [86]. The metabolic rate of an organism is closely related to the body temperature. *R. sibiricus* is distributed at altitudes approximately 2100–3200 m above sea level, where the habitat is at almost the extreme limit of tolerance for cold-blooded animals, and the metabolism level of this species is low. And some studies suggest that *R. sibiricus* is a relatively primitive species among the Hynobiidae [87]–[89]. It is therefore reasonable to infer a slow genomic evolutionary rate for this species. We noticed that different settings of the mutation rate of mtDNA can affect the t1 estimation but not the scenario selection. In this study, because there is no reliable temporal calibration that applies directly to our data, the mutation rate of mtDNA used was the default setting (10^−8^ to 10^−7^ per site per generation), as also used in some studies of other Hynobiidae species [43], [90]. When we set the evolutionary rate of *R. sibiricus* mtDNA to a lower order of magnitude, i.e., 10^−9^ to 10^−8^ per site per generation, the estimated t1 turns out to be greater than 100 generations; however, this estimation does not bias our main conclusion.

Field populations of *R. sibiricus* have declined dramatically during the past three decades, and this sharp decline has clearly had some impact on genetic diversity and population structure, e.g., in the AKS and JMK populations. Under the ABC method, the *N*_e_ of the common ancestral population was roughly two orders of magnitude higher than that of the modern populations, suggesting a relatively large historical population size of this species. The reduction in population size has increased the possibility of inbreeding, as exemplified by the AKS individuals with six toes on the hind limbs. In summary, our results suggest that the *R. sibiricus* populations separated fairly recently (∼120 YBP) as a result of human activities and/or climatic change and that Pleistocene glaciation oscillation may have facilitated intraspecies gene flow rather than enhanced divergence. A low genomic evolutionary rate and elevated inbreeding frequency may also have contributed to the low genetic diversity observed in this species.

### Implications for conservation

Although loss of genetic diversity is recognized as being directly related to population reduction [1], [79], it is important to distinguish whether loss of genetic diversity is the cause of population reduction per se, or whether population reduction is the cause of loss of genetic diversity. Such distinction will provide more effective conservation management for endangered species. However, in our study, it should not make a simplified conclusion regarding such distinction. We conclude that the historical population size of *R. sibiricus* was much larger than the present population, and the causes of extreme genetic uniformity may be a combination of recent and historical factors, including the species' unique population history, slow evolutionary rate at the genomic level and the recent dramatic population decline. The recent decline caused by climatic changes has obviously affected some small populations (such as AKS and JMK), and the trend that “population reduction is the cause of loss of genetic diversity” has become increasingly evident. We should also pay attention to the fact that the geographic range and the population size of *R. sibiricus* have decreased dramatically during the past decade. Because of the high level of genetic homogeneity, *R. sibiricus* populations are prone to reduction of fitness when the environment changes dramatically. The species' adaptations to cold water also make *R. sibiricus* vulnerable to unfavorable environments, particularly the desiccation of wetlands. The rapid extinction of many amphibian species has received great attention in recent years and is attributed mainly to the deterioration of the global climate [14], [15]. The case of *R. sibiricus* suggests that programs to conserve living amphibian species should be accelerated.

Two goals of any conservation program should be to ensure the survival of a species and to maintain its genetic diversity for long-term evolutionary success [91], [92]. However, our study failed to demonstrate a significant level of genetic subdivision between the geographic regions. Therefore, we have insufficient evidence to suggest the division of the population into separate units for conservation management purposes on the basis of the theoretical definition of the evolutionary significant unit. The preservation of extant habitats, support of populations through artificial breeding, and restoration of wetlands or creation of new suitable habitats will probably be the most effective measures for conservation of this species.

## Supporting Information

Text S1 **Approximate Bayesian computation analysis.**
(DOC)

Text S2 **The references of species listed in** **Figure 4** **were listed as follows.**
(DOC)

Table S1 **Primers used in the study of mtDNA and microsatellites.**
(DOC)

Table S2 **The prior distributions of parameters used for description of the three scenarios analyzed via the ABC method.**
(DOC)

Table S3 **Characterization of two polymorphic loci and twenty microsatellite loci with good repeats but no polymorphism in** ***Ranodon sibiricus*** **.**
(DOC)

Table S4 **Bias and precision of parameter estimation under Scenario 1.**
(DOC)
