# Correction: Extremely Low Genetic Diversity Indicating the Endangered Status of *Ranodon sibiricus* (Amphibia: Caudata) and Implications for Phylogeography

**DOI:** 10.1371/journal.pone.0124223

**Published:** 2015-04-09

**Authors:** The PLOS ONE Staff

The GPS coordinates for the JMK population listed in Table 1 are incorrect. The correct coordinates are: N: 44°55’47, E: 80°00’18.

Please see the corrected Table 1 here.

**Table 1:** The habitat and population size data, sampling time, sampling size and sample size of each locality used for (a) mtDNA and (b) microsatellite analysis.

Locality	Discovered time	Coordinates	Area(m^2^)	Altitude (m)	Population size existed	Sampling time	Sampling size	Sample size	
								a	b
SLBZ	1990	N: 44°56’04, E: 80°30’43	40000	2100–2300	>1000	May 2006	20	20	0
SLBZ1#	1991	N: 44°53’27, E: 80°29’45	10000	2041	120	June 2006	20	20	20
SLBZ2#	2006	N: 44°50’01, E: 80°39’10	30000	2250	150	August 2006	20	20	20
JMK	1989	N: 44°55’47, E: 80°00’18	5000	2800	100	June 2006	20	20	20
SAR	2002	N: 44°57’59, E: 80°13’34	15000	2465	120	May 2006	20	20	20
AKS	1996	N: 44°49’53, E: 80°19’26	10000	3200	1500	August 2006	23*	23	20

*Three samples of AKS (21, 22 and 23) obtained by Xiuling Wang in August 2005 had six toes on the hind limbs.
