# Supplementary material for: Extremely Low Genetic Diversity Indicating the Endangered Status of Ranodon sibiricus (Amphibia: Caudata) and Implications for Phylogeography
Source: PLoS One. 2012 Mar 12;7(3):e33378. doi: 10.1371/journal.pone.0033378 (PMC3299782; doi:10.1371/journal.pone.0033378)
Supplement: Text S1 — Approximate Bayesian computation analysis. (DOC) [file pone.0033378.s001.doc]

**Text S1.** Approximate Bayesian computation analysis.

Other parameters of ABC treatment are listed as follows. With regards to parameters for microsatellite and marker, each locus was assumed to own a possible range of 2 contiguous allelic states; other parameters were used the default values. The mean mutation rate (per site per generation) of mitochondrial DNA sequence were set between 1×10^-8^ to 1×10^-7^; other parameters were used the default values. Genetic variation within and between the six populations samples was summarized with several statistics traditionally used in ABC treatments for microsatellite loci: the mean genetic diversity of one sample summary statistics, the *Fst* and classification index of two sample summary statistics. And for mitochondrial DNA sequences, we chose the mean of pairwise differences, variance of pairwise differences and Tajima’s *D* for one sample summary statistics; and mean of pairwise differences (W), mean of pairwise differences (B) and *Fst* for two sample summary statistics.

Using parameter values drawn from the above described, we produced a reference table containing one point five million simulated data sets (500,000 for each scenario). We performed a weighted polychotomous logistic regression to estimate the (relative) posterior probability of scenarios, taking a number of simulated data sets closest to our real data set 30,000 (1%). 95% credibility intervals for the posterior probabilities of scenarios were computed through the limiting distribution of the maximum likelihood estimators. Once the most likely scenario (among the three compared scenarios) was assessed, we used a local linear regression to estimate the posterior distributions of parameters under this scenario. We took the 30,000 (1%) simulated data sets closest to our real data set for the logistics regression, after applying a logit transformation to parameter values. Bias and precision of parameter estimation under the most likely scenario were listed in Table S4.
