# Supplementary material for: Extremely Low Genetic Diversity Indicating the Endangered Status of Ranodon sibiricus (Amphibia: Caudata) and Implications for Phylogeography
Source: PLoS One. 2012 Mar 12;7(3):e33378. doi: 10.1371/journal.pone.0033378 (PMC3299782; doi:10.1371/journal.pone.0033378)
Supplement: Text S2 — The references of species listed in Figure 4 were listed as follows. (DOC) [file pone.0033378.s002.doc]

**Text S2.** The references of species listed in Figure 4 were listed as follows.

(a): 2: Mosquito [1]; 3: Spanish imperial eagle [2]; 4: Basking shark [3]; 5: Bonelli’s Eagle [4]; 6: Green and golden bell frog [5]; 7: Red kite [6]; 8: Tortuguero green turtle [7]; 9: White-headed duck [8]; 10: Steller sea lion [9]; 11: Wolverine [10]; 12: Przewalski’s gazelle [11]; 13: Red panda [12]; 14: Tibetan antelope [13]; 15: Chinese giant salamander [14]; 16: Giant panda [15]; 17: Tibetan gazelle [16].

(b): 2: Florida manatee [17]; 3: Ozark hellbender [18]; 4: Smallmouth salamander [19]; 5: Spotted salamander [20]; 6: Black muntjac [21]; 7: Yellow-spotted night lizard [22]; 8: Chinese soft-shelled turtle [23]; 9: South China tiger [24]; 10: Macedonian crested newt [25]; 11: Sharp-ribbed salamander [26]; 12: Puma [27]; 13: Elliot’s pheasant [28]; 14: Amazon river dolphin [29]; 15: Tiger salamander [30]; 16: Knysna seahorse [31]; 17: Asiatic lion [32].

1. Maia RT, Scarpassa VM, Maciel-Litaiff LH, Tadei WP (2009) Reduced levels of genetic variation in *Aedes albopictus* (Diptera: Culicidae) from Manaus, Amazonas State, Brazil, based on analysis of the mitochondrial DNA *ND5* gene. Genetics and Molecular Research 8(3): 998-1007.

2. Martínez-Cruz B, Godoy A, Negro JJ (2004) Population genetics after fragmentation: the case of the endangered Spanish imperial eagle (*Aquila adalberti*). Molecular Ecology 13: 2243-2255.

3. Hoelzel AR, Shivji MS, Magnussen J, Francis MP (2006) Low worldwide genetic diversity in the basking shark (*Cetorhinus maximus*). Biology Letters. doi: 10.1098/rbsl.2006.0513.

4. Cadahía L, Negro JJ, Urios V (2007) Low mitochondrial DNA diversity in the endangered Bonelli’s Eagle (*Hieraaetus fasciatus*) from SW Europe (Iberia) and NW Africa. Journal of Ornithology 148: 99-104.

5. Burns EL, Eldridge MDB, Crayn DM, Houlden BA (2007) Low phylogeographic structure in a wide spread endangered Australian frog *Litoria aurea* (Anura: Hylidae). Conservation Genetics 8(1): 17-32.

6. Roques S, Negro JJ (2005) MtDNA genetic diversity and population history of a dwindling raptorial bird, the red kite (*Milvus milvus*). Biological Conservation 126: 41-50.

7. Bjorndal KA, Bolten AB, Troeng S (2005) Population structure and genetic diversity in green turtles nesting at Tortuguero, Costa Rica, based on mitochondrial DNA control region sequences. Marine Biology 147: 1449-1457.

8. Muňoz-Fuentes V, Green AJ, Negro JJ, Sorenson MD (2005) Population structure and loss of genetic diversity in the endangered white-headed duck, *Oxyura leucocephala*. Conservation Genetics 6: 999-1015.

9. O’Corry-Crowe G, Taylor BL, Gelatt T, Loughlin TR, Bickham J, et al. (2006) Demographic independence along ecosystem boundaries in Steller sea lions revealed by mtDNA analysis: implications for management of an endangered species. Canadian Journal of Zoology 84: 1796-1809.

10. Chappell DE, Bussche RAVD, Krizan J, Patterson B (2004) Contrasting levels of genetic differentiation among populations of wolverines (*Gulo gulo*) from northern Canada revealed by nuclear and mitochondrial loci. Conservation Genetics 5: 759-767.

11. Lei R, Hu Z, Jiang Z, Yang W (2003) Phylogeography and genetic diversity of the critically endangered Przewalski’s gazelle. Animal Conservation 6: 361-367.

12. Li M, Wei FW, Goossens B, Feng ZJ, Tamate HB, et al. (2005) Mitochondrial phylogeography and subspecific variation in the red panda (*Ailurus fulgens*): implications for conservation. Molecular Phylogenetics and Evolution 36: 78-89.

13. Ruan XD, He PJ, Zhang JL, Wan QH, Fang SG (2005) Evolutionary history and current population relationships of the chiru (*Pantholops Hodgsonii*) inferred from mtDNA variation. Journal of Mammalogy 86: 881-886.

14. Murphy RW, Fu J, Upton DE, Lema TD, Zhao E (2000) Genetic variability among endangered Chinese giant salamanders, *Andrias davidianus*. Molecular Ecology 9: 1539-1547.

15. Zhi L, Warren EJ, Marilyn MR, Naoya Y, Janice SM, et al. (2001) Patterns of genetic diversity in remaining Giant Panda populations. Conservation Biology 15(6): 1596-1607.

16. Zhang FF, Jiang ZG (2006) Mitochondrial phylogeography and genetic diversity of Tibetan gazele (*Procapra picticaudata*): Implications for conservation. Molecular Phylogenetics and Evolution 41: 313-321.

17. Pause KC, Nourisson C, Clark A, Kellogg E, Bonde RK, et al. (2007) Polymorphic microsatellite DNA markers for the Florida manatee (*Trichechus manatus latirostris*). Molecular Ecology Notes 7: 1073-1076.

18. Johnson JR, Faries KM, Rabenold JJ, Crowhurst RS, Briggler JT, et al. (2008) Polymorphic microsatellite loci for studies of the Ozark hellbender (*Cryptobranchus alleganiensis bishopi*). Conservation Genetics 10: 1795-1797. doi: 10.1007/s10592-009-9818-z.

19. Williams RN, Dewoody JA (2004) Fluorescent dUTP helps characterize 10 novel tetranucleotide microsatellites from an enriched salamander (*Ambystoma texanum*) genomic library. Molecular Ecology Notes 4: 17-19.

20. Wieczorek AM, Zamudio KR, King TL, Gjetvaj B (2002) Isolation of microsatellite loci in spotted salamanders (*Ambystoma maculatum*). Molecular Ecology Notes 2: 313-315.

21. Wu HL, Meng K, Zhu GP (2008) Isolation and characterization of microsatellite markers in black muntjac (*Muntiacus crinifrons*). Molecular Ecology Resources 8: 584-586.

22. Sinclair EA, Scholl R, Bezy RL, Crandall KA, Jack WSJR (2006) Isolation and characterization of di- and tetranucleotide microsatellite loci in the yellow-spotted night lizard *Lepidophyma flavimaculatum* (Squamata: Xantusiidae). Molecular Ecology Notes 6: 233-236.

23. Que Y, Zhu B, Rosenthal H, Chang J (2007) Isolation and characterization of microsatellites in Chinese soft-shelled turtle, *Pelodiscus sinensis*. Molecular Ecology Notes 7: 1265-1267.

24. Zhang ZH, Zhang WP, Yue BS, Shen FJ, Zhang L, et al. (2006) Twelve polymorphic microsatellite loci for the South China tiger *Panthera tigris amoyensis*. Molecular Ecology Notes 6: 24-26.

25. Sotiropoulos K, Tsaparis D, Eleftherakos K, Kotoulas G, Legakis A, et al. (2008) New polymorphic microsatellite loci for the Macedonian crested newt, *Triturus macedonicus*, and cross-priming testing in four other crested newt species. Molecular Ecology Resources 8: 1402-1404.

26. Van DE Vliet MS, Diekmann OE, Serrao EA, Beja P (2009) Isolation of highly polymorphic microsatellite loci for a species with a large genome size: sharp-ribbed salamander (*Pleurodeles waltl*). Molecular Ecology Resources 9: 425-428.

27. Kurushima JD, Collins JA, Well JA, Ernest HB (2006) Development of 21 microsatellite loci for puma (*Puma concolor*) ecology and forensics. Molecular Ecology Notes 6: 1260-1262.

28. Jiang PP, Ding P, Fang SG (2006) Isolation and characterization of microsatellite markers in Elliot’s pheasant (*Syrmaticus ellioti*). Molecular Ecology Notes 6: 1160-1161.

29. Gravena W, Hrbek T, Silva VMSD, Astolfi-Filho S, Farias I (2009) Microsatellite loci for population and parentage analysis in the Amazon River dolphin (*Inia geoffrensis* de Blainville, 1817). Molecular Ecology Resources 9: 600-603.

30. Mech SG, Storfer A, Ernst JA, Reudink MW, Maloney SC (2003) Polymorphic microsatellite loci for tiger salamanders, *Ambystoma tigrinum*. Mol Ecol Notes 3: 79-81.

31. Galbusera PHA, Gillemot S, Jouk P, Teske PR, Hellemans B, et al. (2007) Isolation of microsatellite markers for the endangered Knysna seahorse *Hippocampus capensis* and their use in the detection of a genetic bottleneck. Molecular Ecology Notes 7: 638-640.

32. Singh A, Shailaja K, Gaur A, Singh G (2002) Development and characterization of novel microsatellite markers in the Asiatic lion (*Panthera leo persica*). Molecular Ecology Notes 2: 542-543.
