# Supplementary material for: Extremely Low Genetic Diversity Indicating the Endangered Status of Ranodon sibiricus (Amphibia: Caudata) and Implications for Phylogeography
Source: PLoS One. 2012 Mar 12;7(3):e33378. doi: 10.1371/journal.pone.0033378 (PMC3299782; doi:10.1371/journal.pone.0033378)
Supplement: Table S1 — Primers used in the study of mtDNA and microsatellites. (DOC) [file pone.0033378.s003.doc]

**Table S1.** Primers used in the study of mtDNA and microsatellites.

| Primer name | Sequence (5’-3’) | PCR program |
| --- | --- | --- |
| Cyt*b* A | GAATYGGRGGWCAACCAGTAGAAGACCC | PCR reaction was carried out in a 20-μl reaction volume containing 0.2-0.5 µl of genomic DNA, 0.2 mM dNTPs, 2 µl of 10× buffer, 0.2 μM of 12S71R and Cyt*b* A each and 1 µl of Prime polymerase (TaKaRa). Cycling conditions: 94 ℃ for 4 min, followed by 30 cycles of 60 s at 94 ℃, 60 s at 55 ℃ and 90 s at 72 ℃, and a final extension of 10 min at 72 ℃. |
| 12S71R | ATAAGGCTAGGACCAAACCTTT |
| Rsi-5-F | TATGAGCTCCTCCCCAACCTGAGTT | PCR reaction was performed in a 20-μl reaction volume containing 10 ng of genomic DNA, 0.16 mM dNTPs, 2 μl 10× PCR buffer, 0.5 μM of each primer and 1 U Ex Taq DNA polymerase (TaKaRa). Cycling conditions: 94 ℃ for 5 min, followed by 35 cycles of 30 s at 94 ℃, 30 s at 55 ℃ and 60 s at 72 ℃, and a final extension of 10 min at 72 ℃. |
| Rsi-5-R | GCATCTAAGCATCCTCCCT |
| Rsi-17-F | GCAGGCACGGTAGAAATG |
| Rsi-17-R | TGTGGTACAAGGCAAATGGA |
