# Supplementary material for: Extremely Low Genetic Diversity Indicating the Endangered Status of Ranodon sibiricus (Amphibia: Caudata) and Implications for Phylogeography
Source: PLoS One. 2012 Mar 12;7(3):e33378. doi: 10.1371/journal.pone.0033378 (PMC3299782; doi:10.1371/journal.pone.0033378)
Supplement: Table S2 — The prior distributions of parameters used for description of the three scenarios analyzed via the ABC method. (DOC) [file pone.0033378.s004.doc]

**Table S2.** The prior distributions of parameters used for description of the three scenarios analyzed via the ABC method.

| Parameter | Distribution | Min. | Max. | Step |
| --- | --- | --- | --- | --- |
| **Effective population size** |  |  |  |  |
| N1, N4 | uniform | 1 | 500 | 1 |
| N2, N3, N5, N6 | uniform | 1 | 100 | 1 |
| N1a, N2a, N3a, N4a, N5a, N6a | uniform | 1 | 100 | 1 |
| N1b, N2b, N3b, N4b, N5b, N6b | uniform | 10 | 1000 | 1 |
| db | uniform | 5 | 5 | 1 |
| NA | uniform | 10 | 10000 | 1 |
| **Time of events (in generations backward in time)** |  |  |  |  |
| t1 | uniform | 1 | 1000 | 1 |
| t2 | uniform | 2000 | 8000 | 1 |
| t3 | uniform | 10000 | 50000 | 1 |

For description of events considered see also Text S1. As the prior distributions of parameters often overlapped, several conditions were considered. The most important were t3 > t2 > t1, N1a < N1b, N2a < N2b, N3a < N3b, N4a < N4b, N5a < N5b, N6a < N6b.
