# Supplementary material for: Extremely Low Genetic Diversity Indicating the Endangered Status of Ranodon sibiricus (Amphibia: Caudata) and Implications for Phylogeography
Source: PLoS One. 2012 Mar 12;7(3):e33378. doi: 10.1371/journal.pone.0033378 (PMC3299782; doi:10.1371/journal.pone.0033378)
Supplement: Table S3 — Characterization of two polymorphic loci and twenty microsatellite loci with good repeats but no polymorphism in Ranodon sibiricus . (DOC) [file pone.0033378.s005.doc]

**Table S3.** Characterization of two polymorphic loci and twenty microsatellite loci with good repeats but no polymorphism in *Ranodon sibiricus*.

| Marker | Primer sequence(5’-3’) | Sizes range (bp) | Repeat motif | *T*_a_ (°C) Start-end | No.of alleles | GenBankaccession |
| --- | --- | --- | --- | --- | --- | --- |
| Rsi-5 | F: TATGAGCTCCTCCCCAACCTGAGTT  R: GCATCTAAGCATCCTCCCT | 175-185 | CTAT GAG CTAG (CTAT)_12_  (CTAG)_2_(CTAT)_3_ | 55 | 2 | JN863391 |
| Rsi-17 | F: GCAGGCACGGTAGAAATG  R: TGTGGTACAAGGCAAATGGA | 156-160 | (ACTC)_2_ GCAC (ACTC)_13_ | 55 | 2 | JN863392 |
| Rsi-1 | F: ATTAGACTAGAATGCCAGAACAT  R: CCCTAGATGACCTGAACCAG | 441 | [(TG)_3_TT]_2_(TG)_3_(TATG)_2_(TG)_12_(GT)_5_  (AT)_2_(GT)_6_N_7_(TG)_5_C(TG)_12_(GT)_5_TT(GT)_2_ | 55 | 1 | JN863393 |
| Rsi-2 | F: GGCTCTGGCAGAACAC  R: CTTTAGCACGCACTTTG | 253 | (TG)_4_N_2_(TG)_5_N_6_(TG)_2_ N_6_  (TG)_5_ N_13_ (TG)_4_ N_22_ (TG)_4_ | 60-50 | 1 | JN863394 |
| Rsi-4 | F: CACCACAAAGTATTGGTCTC  R: GGCTGCAGTTTAATGACAGTAT | 182 | (GT)_7_AT(GT)_4_N_4_(GT)_2_CT(GT)_13_GC(CT)_2_  (GT)_2_N_10_(GT)_3_N_6_(GT)_2_T_2_(GT)_5_AT(GT)_2_ | 40 | 1 | JN863395 |
| Rsi-11-1 | F: CCTGCTGGTGGAGCGATAT  R: CCTTTGGTGCCTGGAGTT | 278 | (GT)_4_N_3_(TG)_4_N_2_(TG)_2_N_8_  (TG)_2_N_4_(GT)_12_ | 60-50 | 1 | JN863396 |
| Rsi-11-2 | F: AGGAGGAACTCAGCTACCC  R: ACGGCGACTAAGAACACC | 283 | (TGTGTA)_2_CA(TG)_12_ | 60-50 | 1 | JN863397 |
| Rsi-20 | F: GGGCTGCGCCTAACCTCTT  R: GGGCTCCTTGACCTTGCTTTT | 248 | (AC)_23_ | 60-50 | 1 | JN863398 |
| Rsi-21 | F: CAGGGCCTTTTCTTTGTA  R: GGGGCACTTTTCTAGTGTAA | 244 | (GT)_5_N_4_(GT)_2_A(TG)_3_N_2_(GT)_2_N_5_  (GT)_2_N_2_(GT)_9_N_6_(TG)_4_(GT)_10_ | 60-50 | 1 | JN863399 |
| Rsi-24 | F: CAGAATCCCGTGACTCCA  R:CTCTGTACCAATTTATTAAGGCAAC | 234 | (CA)_16_ | 60-50 | 1 | JN863400 |
| Rsi-33 | F: TGGTAACCTCCATAAGAAA  R: TATGTTTGCCACCTGTAG | 236 | (GT)_11_N_3_(GT)_5_ | 60-50 | 1 | JN863401 |
| Rsi-35 | F: CGTGGTTTGTTCTATTCG  R: TCTCCCAACTGAACCTAAA | 232 | (TATC)_10_ | 40 | 1 | JN863402 |
| Rsi-45 | F: AGATGTTTTATTAGCATTTTCG  R: CAGTCTGTTACTTCATGGTTC | 202 | (CTCA)_7_CACA | 60-50 | 1 | JN863403 |
| Rsi-48 | F: GTCCCTTAATTCAAACTATTTGGTC  R: GCTCCATCTGTGATCCCTTCT | 275 | (TG)_7_TT(TG)_3_ | 60-50 | 1 | JN863404 |
| Rsi-49 | F: TTTAACCGTAACTAATAGTT  R: ATAAACTGTTCAGAAATCA | 268 | (TG)_4_AA(TG)_2_TC(TG)_2_N_7_(TG)_10_  AA(TG)_2_N_13_(TG)_3_CG(TG)_3_ | 60-50 | 1 | JN863405 |
| Rsi-50 | F: GTATGCCTGCGGGAGT  R: CAAGTGTTACATGGGACGA | 223 | (GT)_3_AA(GT)_3_N_5_(TG)_13_ | 60-50 | 1 | JN863406 |
| Rsi-52 | F: TTCCAACTCCTACTGTCC  R: GAAGATAAACTCCAGTAAAA | 248 | (CA)_13_ | 60-50 | 1 | JN863407 |
| Rsi-54 | F: CAATTACCCCAGACGT  R: CAGTGGATGAAGCAGAT | 206 | (GGTCT)_5_T_7_ | 60-50 | 1 | JN863408 |
| Rsi-55 | F: GCAAGGAAGCTAGTGAAA  R: GAAAATAGAATAATGGGAGC | 169 | (TG)_6_(CG)_2_(GT)_11_T(TG)_4_N_12_(TG)_3_TA(TG)_4_  (GT)_2_G(GT)_2_N_4_(GT)_2_G(GT)_2_GG(TG)_2_N_3_(GT)_4_ | 55 | 1 | JN863409 |
| Rsi-73 | F: GAGGCAGCACAGTGAGGATTC  R: CCGGGCAGTACCATGATACTT | 187 | (AC)_11_ | 60-50 | 1 | JN863410 |
| Rsi-74 | F: AAGACCTAGCTAGCAGAATCAAT  R: CTTGGAAGGTTGAAGAGGGTG | 163 | AATC CAAT (AATC)_5_ | 60-50 | 1 | JN863411 |
| Rsi-80 | F: CGGCCTCTGTAGAAATATGCT  R: AGAAATGCGCTATGTTAAGTG | 151 | (GTGA)_6_ | 60-50 | 1 | JN863412 |

*T*_a_, locus-specific annealing temperature (touchdown program: start temperature – end temperature, decrease 1 ℃ per cycle, and longer program with 30 cycles at end temperature; other loci’s PCR program steps were the same as Rsi-5 and Rsi-17 except the annealing temperature). Size range refers to the PCR product size at each locus.
