# Supplementary material for: Extremely Low Genetic Diversity Indicating the Endangered Status of Ranodon sibiricus (Amphibia: Caudata) and Implications for Phylogeography
Source: PLoS One. 2012 Mar 12;7(3):e33378. doi: 10.1371/journal.pone.0033378 (PMC3299782; doi:10.1371/journal.pone.0033378)
Supplement: Table S4 — Bias and precision of parameter estimation under Scenario 1. (DOC) [file pone.0033378.s006.doc]

**Table S4.** Bias and precision of parameter estimation under Scenario 1.

|  | Posterior distribution | | | | | Posterior median | | |
| --- | --- | --- | --- | --- | --- | --- | --- | --- |
| Parameter | True value | RRMISE | RMAD | 50% cov. | 95% cov. | MRB | RRMISE | fact2 |
| N1 | 1.500E+001 | 12.708 | 1.794 | 0.506 | 0.962 | 1.350 | 12.708 | 0.752 |
| N2 | 8.400E+001 | 5.444 | 1.639 | 0.484 | 0.968 | 1.143 | 5.444 | 0.736 |
| N3 | 5.100E+001 | 6.192 | 1.832 | 0.526 | 0.954 | 1.341 | 6.192 | 0.716 |
| N4 | 4.670E+002 | 7.857 | 1.454 | 0.560 | 0.964 | 1.006 | 7.857 | 0.764 |
| N5 | 1.000E+000 | 6.226 | 1.899 | 0.510 | 0.956 | 1.412 | 6.226 | 0.712 |
| N6 | 5.000E+000 | 5.736 | 1.643 | 0.492 | 0.968 | 1.129 | 5.736 | 0.736 |
| t1 | 4.470E+002 | 3.646 | 0.931 | 0.544 | 0.980 | 0.501 | 3.646 | 0.772 |
| NA | 2.717E+003 | 6.533 | 1.851 | 0.494 | 0.968 | 1.413 | 6.533 | 0.678 |
| Mμmic_A | 7.200E-004 | 1.303 | 0.770 | 0.508 | 0.960 | 0.385 | 1.303 | 0.800 |
| Mμseq_M | 2.100E-008 | 1.301 | 0.767 | 0.528 | 0.970 | 0.328 | 1.301 | 0.808 |

Extract of the output of *DIY ABC*/option ”Compute bias and mean square error” with scenario 1 and parameter values identical to those used to simulate the example data set. Measures relative to the posterior distribution of parameters are the relative square root of the mean integrated square error (RRMISE), the relative mean absolute deviation (RMAD) and the proportion of times where the true value is within the 50% and 95% credibility intervals (*50% cov* and *95% cov*., respectively). Precision measures are also given for the posterior median as a point estimate: the mean relative bias (MRB), the relative square root of the mean square error (RRMSE) and the proportion of times the posterior median is between the half and the double of the true value (*fact2*).
